# Modern Aspects of Malaria Problems in Peace and War

**Published:** 1919

**Authors:** 


***Modern Aspects of Malaria Problems in Peace and War.*** By Frederick L. Hoffmann. *Southern Medical Journal*, August, 1918.

A case of malaria may be the mildest form of parasitical infection, quite unknown or unobserved by the person affected. It may be a latent case, or one that is quite harmless, or one of the most pernicious varieties. It will probably always be a difficult matter to secure entirely accurate and complete returns of malaria cases by whatever system or method of reporting may be adopted.

A positive blood-index is of course entirely conclusive, but a negative index is not necessarily evidence of the absence of infection. The fact that the absence of malaria plasmodium in the peripheral circulation fails occasionally to disclose the presence of the disease is not sufficient to seriously disturb the accuracy of the conclusions arrived at.

In Bolivar County a well-considered plan for quinine immunization has been put into effect. Whether that effort will prove entirely successful is, for the time-being, an open question, but the evidence thus far available seems to prove quite conclusively that the percentage of parasitical infections is being materially reduced.

In Arkansas, at Crossett and Hamburg, reliance is placed exclusively upon drainage, screening, etc. Mosquitoes have been largely exterminated, and very considerable reduction in the incidence of malaria has followed within a relatively short period of time.

On account of the war, vast bodies of northern troops have been placed in southern cantonments with the practical certainty of extensive exposure to the risk of malaria infection. It is, therefore, of the utmost importance that effective control measures should be applied to the extra-cantonment areas in the southern States where malaria is known to prevail.

Every doctor throughout the south should be required to report cases of malaria in precisely the same manner as cases of acute infectious diseases or tuberculosis, poliomyelitis, etc., are reported.

In exact proportion as we improve our methods of reporting infectious or transmissible diseases, including malaria, we shall lay the foundation for more successful eradication measures than have heretofore been adopted and found useful in usual practice.

